# Analysis of Direct-to-Consumer Marketing of Platelet-Rich Plasma for Erectile Dysfunction in the US

**DOI:** 10.1001/jamanetworkopen.2022.14187

**Published:** 2022-05-26

**Authors:** Gary K. Shahinyan, James M. Weinberger, Robert H. Shahinyan, Shangyang C. Yang, Jesse N. Mills, Sriram V. Eleswarapu

**Affiliations:** 1 University of Colorado School of Medicine, Aurora; 2 Division of Andrology, Department of Urology, David Geffen School of Medicine at the University of California, Los Angeles; 3 Department of Urology, the University of Caliornia Davis School of Medicine, Sacramento

## Abstract

This cross-sectional study investigates costs, treatment protocols, and clinician credentials of platelet-rich plasma injection therapy for erectile dysfunction in the US.

## Introduction

Platelet-rich plasma (PRP) has become a part of the armamentarium of various specialties, with evolving indications in dermatology, orthopedics, and other fields. Recently, the use of PRP has expanded into the treatment of erectile dysfunction (ED), despite guidelines from professional societies, such as the American Urological Association, that classify PRP as investigational and not to be provided for payment.^1^ Undeterred by the absence of high-quality evidence, clinics offering PRP injections for ED (often termed the *Priapus shot* or *P shot*) have proliferated through a combination of direct-to-consumer advertising and a market of men looking for novel cures. The increase in the use of PRP injections for ED echoes an earlier era of men’s health fads: the proliferation of direct-to-consumer marketing for treatment of low testosterone from 2001 to 2011, during which time testosterone prescriptions tripled in the US, often among men without a clear indication for testosterone therapy.^2,3^ We sought to characterize the landscape of PRP injection therapy for ED in the US by using secret shopper methods to investigate costs, treatment protocols, and clinician credentials.

## Methods

We conducted a cross-sectional study using a secret shopper approach to characterize the offerings of PRP injections for ED across 8 of the most populous metropolitan areas in the US. The secret shopper approach is a market research method that involves using a script to inquire about products and pricing in service industries. Using internet search, we identified clinics offering PRP for ED in Atlanta, Georgia; Boston, Massachusetts; Dallas, Texas; Houston, Texas; Los Angeles, California; New York, New York; Philadelphia, Pennsylvania; and Washington, DC. Search queries included *PRP for erectile dysfunction in [X]*; *PRP for ED in [X]*; *Priapus shot in [X]*; and *P shot in [X]* (where [X] represents the metropolitan area). Clinics were contacted via telephone from August 1, 2020, to September 30, 2021, by 4 of us (G.K.S., J.M.W., R.H.S., and S.C.Y.) using a standardized script to request information on pricing, protocols, and clinician credentials. Standardization was used to minimize bias. Clinics that did not respond to 3 contact attempts were excluded. Descriptive statistics, including mean values and ranges, were calculated. This study followed the Strengthening the Reporting of Observational Studies in Epidemiology (STROBE) reporting guideline and was granted exemption from institutional review board evaluation by the University of California, Los Angeles, because the study involved anonymous collection of publicly available information without intervention and without identification of any individuals or groups of individuals.

## Results

We identified a sample size of 109 clinics offering PRP injections for ED; data on pricing and treatment duration were available for 90 clinics (83%). The Figure shows the types of clinicians offering PRP injections for ED. Ten clinicians (9%) were urologists, while 24 (22%) were not physicians. The Table illustrates the pricing of PRP injections for ED across 8 metropolitan areas. The mean (SD) price was $1507 ($388). Treatment duration ranged from 1 session to an indefinite number of injections, with no standardized protocol or effectiveness assessment.

**Figure.**
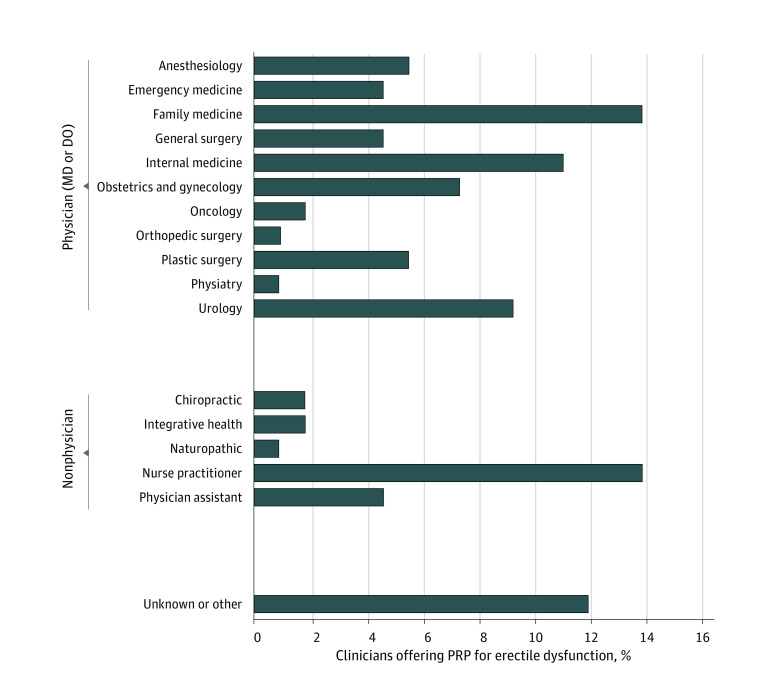
Distribution of Clinician Types Offering Platelet-Rich Plasma (PRP) for Erectile Dysfunction in 8 Major Metropolitan Areas in the US

**Table.** Number of Clinics and Price per Treatment of Platelet-Rich Plasma Injections for Erectile Dysfunction in 8 Major Metropolitan Areas in the US

Metropolitan area	No. of clinics	Mean price per injection, $	Price range, $
Atlanta, GA	9	1561	600-1900
Boston, MA	15	1566	500-2100
Dallas, TX	7	1500	600-1900
Houston, TX	15	1576	1200-1900
Los Angeles, CA	20	1367	650-2500
New York, NY	9	1522	1100-1900
Philadelphia, PA	10	1505	900-2500
Washington, DC	5	1580	1200-1900

## Discussion

Despite a paucity of evidence for its use, PRP injections for the treatment of ED are offered at substantial cost, with no standardized protocol or duration of treatment, and by a considerable number of nonphysicians as well as physicians with no formal training in male sexual dysfunction, such as gynecologists. These findings suggest that guideline-nonconformant care has been driven by the consumerization of sexual health.

Advertising is associated with patient demand, particularly in men’s health. Direct-to-consumer platforms tout consumer convenience, but these companies have been shown to omit appropriate medical evaluation, which may lead to patient harm.^4^ Regardless, there is a burgeoning market for experimental ED therapies, such as PRP injections, low-intensity shockwave therapy, and autologous stem cell injections. As men encounter health information increasingly through social media, physicians trained in male sexual health must serve as stewards of patient education.

A limitation of this study is the selective focus on large metropolitan areas, which may not be representative of smaller or rural areas. A strength of this study is the use of a secret shopper method, which allowed for direct canvassing of practices to obtain firsthand data.
