# Errors in Text

**DOI:** 10.1001/jamanetworkopen.2022.45075

**Published:** 2022-11-04

The Research Letter, “Analysis of Direct-to-Consumer Marketing of Platelet-Rich Plasma for Erectile Dysfunction in the US,”^1^ published in *JAMA Network Open* on May 26, 2022, included information that requires correction. The article stated that “PRP injections for the treatment of ED are offered…with no standardized protocol or duration of treatment….” The authors were unaware, however, that PRIAPUS SHOT and P-SHOT are federally registered service marks, or that the owner of these marks has licensed and trained physicians to follow protocols in their use. The article has been corrected.^1^

## References

[1] Shahinyan GK, Weinberger JM, Shahinyan RH, Yang SC, Mills JN, Eleswarapu SV. Analysis of direct-to-consumer marketing of platelet-rich plasma for erectile dysfunction in the US. JAMA Netw Open. 2022;5(5):e2214187. doi:10.1001/jamanetworkopen.2022.14187 35616943 PMC9136622

